# Downregulation of microRNA-300-3p promotes steatosis-to-MASH progression by regulating STX17

**DOI:** 10.3389/fphar.2026.1804415

**Published:** 2026-04-28

**Authors:** Shuixian Du, Xueru Chu, Shousheng Liu, Zhenzhen Zhao, Zhanglong Yin, Yongning Xin, Quanjiang Dong

**Affiliations:** 1 Department of Infectious Disease, Qingdao Municipal Hospital, Dalian Medical University, Qingdao, China; 2 School of Medicine and Pharmacy, Ocean University of China, Qingdao, China; 3 Clinical Research Center, Qingdao Municipal Hospital, Qingdao University, Qingdao, China; 4 Qingdao Medical College, Qingdao University, Qingdao, China; 5 Department of Gastroenterology, Central Laboratories, Qingdao Municipal Hospital, Dalian Medical University, Qingdao, China

**Keywords:** metabolic dysfunction-associated fatty liver disease (MAFLD), metabolic dysfunction-associated steatohepatitis (MASH), MicroRNA - 300 - 3p, simple steatosis (MAFL), STX17

## Abstract

**Background & aims:**

Metabolic dysfunction-associated fatty liver disease (MAFLD) ranges from simple steatosis (MAFL) to metabolic dysfunction-associated steatohepatitis (MASH). With the complex etiological factors associated with MAFLD, the molecular mechanism underlying the progression from steatosis to MASH has not been fully elucidated, resulting in a limited number of effective therapeutic interventions.

**Methods:**

We examined the profiling changes of MicroRNAs (miRNAs) *in vivo* and *in vitro* experiments and miR - 300 - 3p was identified as a crucial checkpoint in this process in the current experiments. The present studies were carried out to explore the role of miR - 300 - 3p in the advancement of MASH. Bioinformatic analysis, RNA - Sequencing, and dual - luciferase reporter assays were used to investigate the might target of miR - 300 - 3p.

**Results:**

We investigated the profiling changes of miRNA and identified miR - 300 - 3p is low expressed in mouse experiments and HepG2 cells. *In vitro* loss of function study revealed that suppression of miR - 300 - 3p in steatotic HepG2 cells could induce obvious inhibit cell autophagy, promote cell apoptosis, cause lipid accumulation, hepatic inflammation and injury, which are the primary characteristics within the pathology of MASH. STX17 was a predicted target gene of miR-300-3p through RNA-Sequencing, bioinformatic analysis and dual-luciferase reporter assays. Downregulation miR-300-3p was able to downregulate STX17 to promotes steatosis-to-MASH progression. In contrast, transduction of miR - 300 - 3p and over-STX17 inhibit steatosis-to-MASH progressionin. The results was inconsistent with canonical miRNA biology predicts, which indicated that the regulation of miR - 300 - 3p on STX17 might has context-dependent and involve indirect mechanism, feedback loop or multi-level control. Potentially clinically significant, the miR - 300 - 3p/STX17 regulatory axis has also been confirmed in human plasma. Expression level of miR - 300 - 3p and STX17 was reduced in plasma of MAFLD patients. Their expression levels were closely related to liver biochemical indicators and lipid profile indicators.

**Conclusion:**

Our research results highlight the function of the miR - 300 - 3p/STX17 regulatory axis in the progression from steatosis to MASH. This suggests that either the supplementation of miR - 300 - 3p or STX17 may provide a promising therapeutic strategy for treating MASH in hepatocytes. The underlying mechanism through which miR - 300 - 3p regulates STX17 requires further investigation.

## Introduction

Metabolic dysfunction-associated fatty liver disease (MAFLD) impact up to 38% of the adult population worldwide, which has become the most widespread chronic liver disorder (Wong et al., 2023; Targher et al., 2025). MAFLD is a multi-stage hepatic lesion syndrome that may progress from simple steatosis (MAFL) to metabolic dysfunction-associated steatohepatitis (MASH), and ultimately to cirrhosis and hepatocellular carcinoma (HCC) (Diehl and Day, 2017; Zhang et al., 2020). MAFLD has emerged as one of the most prevalent etiologies for liver transplantation (Zhang et al., 2020; Pais et al., 2016; Younossi et al., 2016). The global prevalence of MAFLD is substantial, exerting a notable influence on healthcare providers. MAFLD often results from excess visceral fat due to physical inactivity and poor diet. It frequently co-exists with other metabolic and obesity-related conditions like dyslipidemia, type 2 diabetes (T2DM), metabolic syndrome, and hypertension, significantly increasing the risk of cardiovascular disease (CVD) (Anwar et al., 2023).

As a disorder of hepatic lipid metabolism, the distinctive feature of MAFLD is the build - up of lipid droplets inside the cell, which are organelles with the capacity to store both inert and toxic lipids that may initiate the activation of stress pathways (Targher et al., 2025; Mashek et al., 2015). Impaired lipid-droplet remodeling decreases very-low-density lipoprotein secretion and hampers mitochondrial lipid oxidation, thereby contributing to liver disease progression, inflammatory cell death, fibrogenesis activation, and carcinogenesis (Moretti et al., 2024; Loomba et al., 2021; Valenti et al., 2016).

Recent studies have verified the existence of a“multiple - hits”mechanism involving multiple factors in the pathogenesis and advancement of MAFLD. These factors trigger a series of parallel events through complex interactions between host genetic characteristics, environmental risk factors, and gut microbiota (Anwar et al., 2023; Hur et al., 2015). Unfortunately, due to insufficient understanding of the molecular mechanisms, there is still a lack of effective therapeutic targets for MAFLD and disease progression.

MicroRNAs (miRNAs) have garnered substantial attention owing to their regulatory function in gene expression (Zhang et al., 2015; Yang et al., 2025). MiRNAs belong to a category of small non-coding RNAs. Many evidence shown that miRNAs have a function in controlling the expression of genes related to lipid metabolism, inflammation, cell proliferation and fibrosis (Carpi et al., 2024; Mohr et al., 2021). In recent years, numerous studies have indicated that miRNAs contribute to the pathogenesis of MAFLD/MASH by regulating the development and progression of the disease at different levels (Chrysavgis and Cholongitas, 2024). They are the most extensively studied epigenetic modifications in MAFLD, and the role in HCC has also been reviewed in detail (Cotter and Rinella, 2020). In studies related to miRNAs in MAFLD and the progression, miR-34a, miR-122, and miR-21 are the most well-studied,which have also been proven to be closely associated with MAFLD (Leti et al., 2015). The utilization of synthetic mimics or inhibitors to modulate these miRNAs presents a hopeful therapeutic method which is garnering growing attention on both fundamental and medical research.

Currently, some research has indicated that miR - 300 - 3p participates in controlling cell proliferation, migration, and invasion in various cancers, including glioblastoma, breast cancer, and liver cancer (BI et al., 2020). Additionally, studies suggest that miR - 300 - 3p alleviates hypoxia/reoxygenation-induced cardiomyocyte damage by modulating autophagy (Yan -na et al., 2022), can affect transient Ischaemic Attack in Rats (Li et al., 2018) and it can also influence the occurrence of obesity by regulating lipid metabolism (Liang et al., 2023).

In our preliminary studies, we observed a decrease in the expression of miR - 300 - 3p in both MAFLD cell models and mouse models, but the specific mechanism still leftovers unclear. This research endeavors to elucidate the function of miR - 300 - 3p in hepatic lipid metabolism and hepatocyte inflammatory response via in experiments. We will explore the regulatory pathway of miR - 300 - 3p and investigate the role and mechanism of miR - 300 - 3p in promoting the development of MAFLD.

## Materials and methods

### Cell culture and treatment

The human hepatocyte cell line HepG2 was procured from the American Type Culture Collection (ATCC, United States) and cultivated in a humidified incubator maintained at 37 °C with a 5% CO_2_atmosphere. The culture medium employed was Dulbecco’s Modified Eagle Medium (DMEM) (Gibco, United States), supplemented with 1% penicillin - streptomycin solution (Invitrogen, United States) and 10% fetal bovine serum (FBS) (Gibco, United States). To create the *in vitro* MAFLD cell model, HepG2 cells were bred for 24 h either in the absence or presence of 1 mM free fatty acids (FFAs, with a volume ratio of oleic acid to palmitic acid of 2:1), and subsequently employed for the designated assays.

### Experimental animals

Eight-week-old male C57BL/6 mice were purchased from Jinan Pengyue (Jinan, China) and located in a specific pathogen-free animal facility. They were split into a control group that was fed a normal diet and an obesity group that was fed a high - fat diet (HFD; n = 6 each) for 16 weeks. Body weight was recorded weekly during the model construction process. Following the experiment, the mice were humanely sacrificed after a 6 - hour fasting period. Following the anesthesia of mice, blood was collected from the orbital region. Subsequently, the mice were dissected to observe and document the states of various organs, after which the liver was completely isolated. The liver was rinsed with physiological saline, photographed, and weighed. We collected liver tissues from HFD-induced MAFLD mice and control mice, and detected the relative concentrations of miR-300-3p in each group by qRT-PCR. All animal experiments were conducted in compliance with the guidelines of the Medical Laboratory Animal Care Committee of Qingdao Municipal Hospital (Qingdao, China).

### Cell transfection

To conduct experiments on the gain- and loss-of-function of miR - 300 - 3p, miR - 300 - 3p mimics (miR10000449-1-5; Ribobio, Guangzhou, China) or a miR - 300 - 3p inhibitor (miR20000449-1-5; Ribobio, Guangzhou,China) were used to transfect HepG2 cellsor, along with the relevant scrambled control groups (miR1N0000001-1-5, Ribobio, miR2N0000002-1-5, Ribobio). Using the RiboFECTTMCP as transfection ReagentsReagent (C10511-05; Ribobio, Guangzhou, China). Cells were cultured in 12-well plates (Corning) and underwent transfection when reaching a confluency of 80%–90% with Opti-MEM reduced serum medium (GIBCO).

### Oil Red O staining and triglyceride (TG) determination

Cells with a confluence rate of approximately 80%–90% were seeded, followed by transfection within 24 h. Subsequently, the cells were treated with free fatty acids (FFAs) for 24 h to establish a MAFLD cell model. The preparation protocol of FFAs was referenced from the method described by the article (Zhang et al., 2020). Palmitic acid and oleic acid were fully diluted with 1% fat-free bovine serum albumin (BSA) and then mixed at a molar ratio of 1:2. The resulting mixture of FFAs was added to complete medium to prepare a lipid supplement medium with a final concentration of 0.5 mM. Intracellular lipid droplets were stained with Oil Red O staining kit (Solarbio, China), and intracellular triglyceride (TG) content was quantitatively detected using a triglyceride detection kit (Nanjing Jiancheng Bioengineering Institute, China) (Zhang et al., 2020).

### Apoptosis assay of HepG2 cells

Flow cytometry was used to evaluate the apoptosis of HepG2 cells. Following the digestion of cells using trypsin, conduct two rounds of washing with phosphate-buffered saline (PBS). Then, the cells were resuspended in 1× binding buffer to prepare a density of 1 × 10^6^ cells/mL. 100 μL of this cell suspension was taken in a flow tube, and 5 μL of Annexin V (BD Biosciences) and 5 μL of 7-aminoactinomycin D (7-AAD, BD Biosciences) were added. After vortexing and mixing them thoroughly, they were incubated in the dark at room temperature for 15 min. Subsequently, 400 μL of binding buffer was added, and the sample in the tube was gently mixed. Apoptosis analysis was performed using a flow cytometer (BD Biosciences). Each experiment was repeated three times to support statistical analysis.

#### Real-time polymerase chain reaction (PCR)

Total ribonucleic acid (RNA) was extracted from HepG2 cells using Trizol reagent (Invitrogen: 15,596–026, United States) according to the instructions. Complementary deoxyribonucleic acid (cDNA) synthesis was carried out using a cDNA synthesis kit (Thermo Fisher: K1622, United States).

The primers employed for the PCR amplification of fragments encompassing Caspase-3, LC3, P62, FASN, SREBP1c, STX17, IL-2, IL-6, IL-8, TNF-α, Bcl-2 and Bax were synthesized by Nanjing Sipu Kim Technology Co. Ltd. The primer pairs utilized in this research are presented in the subsequent table (Table 1).

**TABLE 1:** Primer sequence for qRT-PCR in this study.

Gene	Primer/Sequences of product ID of primer
Caspase-3	F:5′-TGCTATTGTGAGGCGGTTGT-3′
	R:5′-TCACGGCCTGGGATTTCAAG-3′
LC3	F:5′-AGTTCCTTGTACCTGACCATGTC-3′
	R:5′-ATACACCTCTGAGATTGGTGTGG-3′
P62	F:5′-TGAGGAACAGATGGAGTCGGATA-3′
	R:5′-CATCTGTAGGGACTGGAGTTCACG-3′
SREBP1c	F:5′-CATGGACGAGCCACCCTTC-3′
	R:5′-GCCGACTTCACCTTCGATGT-3′
STX17	F:5′-CCGGCGGGAGGTTTTTCTAT-3′
	R:5′-ATAGCTGGTTCAAGACGGCG-3′
IL-2	F:5′-TGTCACAAACAGTGCACCTACT-3′
	R:5′-GCATCCTGGTGAGTTTGGGA-3′
IL-6	F:5′-GATGTCTGAGGCTCATTCTGCC-3′
	R:5′-CCAGGGCTAAGGATTTCCTG-3′
IL-8	F:5′-CACTGCGCCAACACAGAAAT-3′
	R:5′-TTCTCAGCCCTCTTCAAAAACTTC-3′
TNF-α	F:5′-AAAACAACCCTCAGACGCCA-3′
	R:5′-TCCTTTCCAGGGGAGAGAGG-3′
Bcl-2	F:5′-AAAAATACAACATCACAGAGGAAGT-3′
	R:5′-GTTTCCCCCTTGGCATGAGA-3′
Bax	F:5′-TGATGGACGGGTCCGGG-3′
	R:5′-CAAAAGGGCCCCTGTCTTCA-3′
FASN	F:5′-CCTGGCTGCCTACTACATCG-3′
	R:5′-CACATTTCAAAGGCCACGCA-3′

The PCR amplification regimen was as shown blow: Pre-denaturation was conducted at 95 °C for 10 min, followed by 35 cycles of denaturation at 95 °C for 15 s, annealing at 58 °C for a duration of 60 s, and an extension step at 72 °C for 30 s. The final extension is performed for 10 min at 72 °C. Gene quantification is performed using the comparative threshold cycle (CT) method.

### Western blotting

HepG2 cells with miR-300-3p overexpression or inhibition were cultured in 12-well plates using DMEM complete medium. When the density of the cells reaches 80%–90%, wash the cells three times with PBS, and then lyse them with protein lysis buffer on ice for 30 min. Centrifuge the protein lysis buffer at 12,000×g for 10 min, and collect the supernatant. Subsequently, each protein sample was subjected to electrophoresis on a sodium dodecyl sulfate-polyacrylamide gel electrophoresis (SDS-PAGE) gel at different concentrations. Conduct the film transfer operation subsequent to electrophoresis. Subsequently, the membrane was blocked with 5% bovine serum albumin (BSA) for 60 min at room temperature and then incubated with primary antibodies, including anti-P62 antibody (Species: rabbit; Company: CST; Catalog: Q13501) (1:1000), anti-SREBP-1c (Species: rabbit; Company: Absin; Catalog: abs131802) (1:500), anti-Bcl-2 (Species: rabbit; Company: Absin; Catalog: abs131701) (1:1000), anti-LC3 (Species: rabbit; Company: CST; Catalog: Q9H492) (1:1000), anti-FASN (Species: rabbit; Company: Absin; Catalog: abs135626) (1:1000), anti-TNF-α (Species: rabbit; Company: Absin; Catalog: abs149748) (1:1000), anti-Caspase-3 (Species: rabbit; Company: Proteintech; Catalog: 19677-1-AP) (1:1000), anti-Bax (Species: rabbit; Company: Bimaka; Catalog: A5131) (1:1000), anti-STX17 (Species: rabbit; Company: Proteintech; Catalog: 17815-1-AP) (1:1000), and anti-Tubulin (Species: rabbit; Company: Absin; Catalog: abs131994) (1:1000). The membrane was incubated with primary antibody at 4 °C for at least one night, and then washed three times with PBS containing 0.1% Tween-20 (PBST) for 10 min every time. Subsequently, incubate the membrane with secondary antibody at room temperature for 1 h, followed by three washes with PBST. Protein bands were visualized using ECL reagent (Millipore, United States). Employing Tubulin as an internal control, the signal intensity of each band was analyzed using ImageJ software.

### Immunofluorescence

Cells were fixed with 4% paraformaldehyde for 15 min at room temperature, permeabilized with 0.1% Triton X-100, and then the HepG2 cells and spheroids were incubated with primary and then secondary antibodies. Subsequently, the nuclei were stained with 4^’^,6-Diamidino-2-phenylindole (DAPI). Images of HepG2 cells and spheroids were captured under a fluorescence microscope (Nikon, Japan).

### Identification of target genes for miR - 300 - 3p

Possible target genes of miR - 300 - 3p were forecasted with online bioinformatics tools, including miRDB, miRTarBase, miRanda, and TargetScan. A Venn diagram was constructed using VennDiagram software to identify the overlapping target genes. Kyoto Encyclopedia of Genes and Genomes (KEGG) enrichment analysis of the target genes was performed utilizing the clusterProfiler package in RStudio software, and a network metabolic pathway map was generated. Genes involved in autophagy-related pathways were screened, and miR - 300 - 3p within the 3′ untranslated region (3′UTR) were predicted. Based on the database scores and the functional annotations of each gene, STX17 was recognized as a potential target gene of miR - 300 - 3p, which was further verified by quantitative real-time PCR (qRT-PCR), Western blot analysis and Dual-luciferase reporter.

### Dual-luciferase reporter

The gene sequence of STX17 was cloned and then inserted into the pmirGLO. The eukaryotic vectors expressing STX17 were designated as pmirGLO STX17 - WT and pmirGLO STX17 - MUT. To investigate the affinity of the transcription factor STX17 for the miR - 300 - 3p promoter sequences, a recombinant plasmid lipo2000, encompassing the miR - 300 - 3p promoter sequences or forecasted binding sites, was built and transferred into 293T cells along with pmirGLO STX17 - WT or pmirGLO STX17 - MUT. 293T cells were cultivated in a constant temperature and humidity incubator at 37 °C and 5% CO_2_, using a medium containing 10% fetal bovine serum (FBS) and 1% penicillin-streptomycin. Cells are seeded into a 24 - well plate at a density of 5 x 10^4^ cells per well. For each well, cells were transfected in accordance with the manufacturer’s protocol. 24–48 h post-transfection, the culture medium was aspirated. Cells were gently washed once with 1x phosphate-buffered saline (PBS) and lysed with 100 µL of 1x Passive Lysis Buffer (PLB, Promega) per well with gentle shaking for 10–15 min at room temperature. Luciferase activity was measured successively via the Dual-Luciferase Reporter Assay System (Promega, FR201-01-V2).

### Population study subjects

This study was approved by the Human Research Ethics Committee of Qingdao Municipal Hospital (Qingdao, China). All the participants had Northern Han Chinese ethnicity, and the study was carried out in accordance with the principles of the Declaration of Helsinki and its revisions (Rickham, 1964; Du et al., 2016). This study included a total of 30 Chinese adult patients (both male and female) diagnosed with MAFLD through liver biopsy, as well as 30 age- and gender-matched healthy controls between January 2024 and June 2025. Clinical data of the patients and healthy controls were collected from Qingdao Municipal Hospital. MAFLD and MAFLD Activity Score (MAS) were diagnosed through standard clinical evaluations in accordance with the criteria of the Asian Pacific Association for the Study of the Liver (APASL) (Eslam et al., 2025). Subjects with infectious diseases, other liver diseases, diabetes mellitus, concurrent major renal diseases or cardiac disorders were excluded from the study.

### Baseline biochemical and demographic analyses

A standardized research survey was employed to acquire fundamental clinical information (including name, height, weight, age, gender, smoking status, and hypertension status). Blood specimens were collected from each participant after a 12-h overnight fast. Serum levels of aspartate aminotransferase (AST), alanine aminotransferase (ALT), TG, total cholesterol (TC), low - density lipoprotein (LDL), high - density lipoprotein (HDL), hyaluronic acid (HA), platelet count (PLT) and fasting plasma glucose (FPG) were determined via routine enzymatic assays. Fibroscan is used for detecting the degree of steatosis and liver fibrosis in patients. The pathological report and MASH scoring are reviewed by two pathologists.

### Statistical analyses

Quantitative data are presented as mean ± standard deviation when following a normal distribution. Experiments were conducted with three separate replications. Experimental data were processed using GraphPad Prism10 software, with independent samples t-tests employed for inter-group comparisons. Pearson correlation analysis was employed to evaluate two-parameter correlations. Statistical significance was set as P < 0.05.

## Results

### Downregulation of miR - 300 - 3p promotes FFAs-induced lipid accumulation and hepatic inflammation in HepG2 cells

**FIGURE 1:**
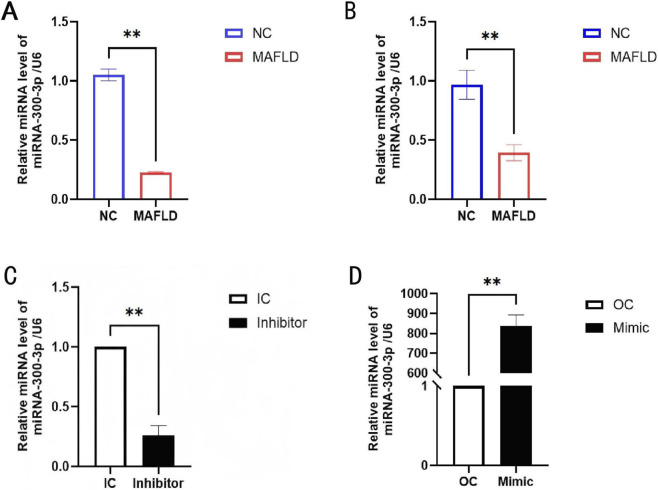
MiR-300-3p was decreased in the livers of MAFLD mice and FFA-induced HepG2 cells. **(A)** MiR-300-3p was decreased in the livers of MAFLD mice. **(B)** MiR-300-3p was downregulated in the FFA-induced HepG2 cells. **(C)** MiR-300-3p was downregulated in HepG2 cells successfully. **(D)** MiR-300-3p was overexpressed in HepG2 cells successfully. Data in **(A–D)** are means±SDs (n = 3). *P < 0.05, **P < 0.01. NC, Healthy control; OC, over-expression control; IC, inhibition control.

In the current research, we successfully established a MAFLD mice model by feeding 8-week-old male C57BL/6 mice a high-fat diet (HFD) for 16 weeks we explored the profiling alterations of MicroRNAs in experiments and resolved that miR - 300 - 3p is shown at a low level in the livers of MAFLD mice and FFA - induced HepG2 cells (Figures 1A,B).

**FIGURE 2:**
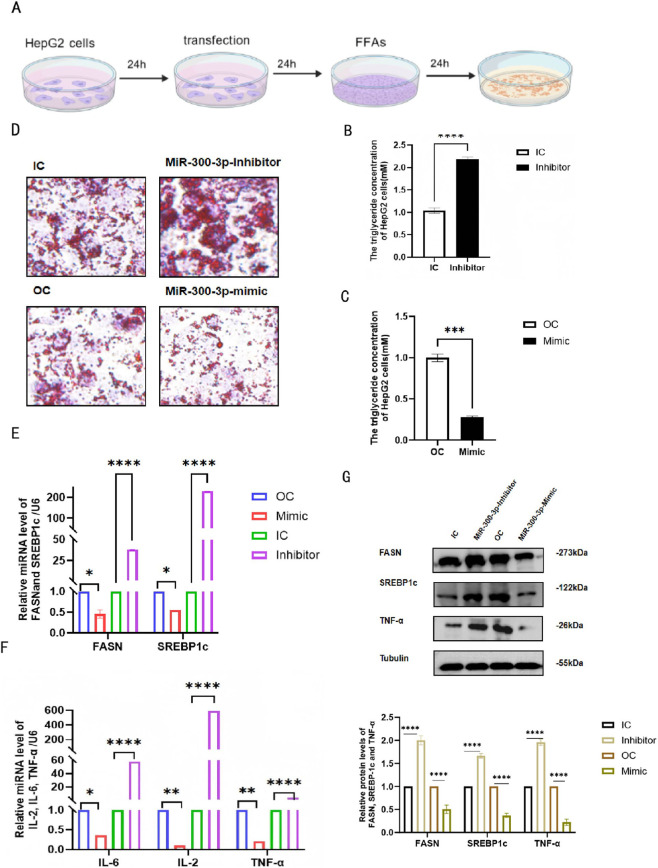
Downregulation of miR-300-3p promotes FFA-induced lipid accumulation and hepatic inflammation in HepG2 cells. **(A)** The experimental flow of the MAFLD cell model construction and miR-300-3p mimics, miR-300-3p inhibitors and as well as corresponding scrambled controls transfection in HepG2 cells. **(B,C)** Intercellular TG contents in miR-300-3p -inhibited and -overexpressing in HepG2 cells. **(D)** Oil Red O staining (400×) and the relative areas of lipid droplets in miR-300-3p-inhibited and -overexpressing in HepG2 cells. **(E,F)** mRNA levels of FASN, SREBP-1c, IL-6, IL-2 and TNF-αin miR-300-3p -inhibited and -overexpressing in HepG2 cells. **(G)** Protein levels of FASN, SREBP-1c and TNF-α in miR-300-3p -inhibited and -overexpressing in HepG2 cells. Data in **(B,C)** and **(E–G)** are means±SDs (n = 3). *P < 0.05, **P < 0.01, ^***^P < 0.001, ^****^P < 0.0001. OC, over-expression control; IC, inhibition control.

The experimental procedure for constructing the MAFLD cell model and subsequently transfecting cells with miR - 300 - 3p mimics, miR - 300 - 3p inhibitors, and corresponding scrambled controls is depicted in (Figure 2A). MiR - 300 - 3p was downregulated or overexpressed in HepG2 cells successfully (Figures 1C,D). In miR - 300 - 3p-inhibited HepG2 cells, the intracellular lipid droplets, triglyceride (TG) contents, and inflammatory factors exhibited a significant increase. Conversely, in miR - 300 - 3p - overexpressing HepG2 cells, these parameters decreased significantly (Figures 2B–D). At the mRNA and protein levels, the expression of FASN, SREBP - 1c, and TNF -ɑ was notably elevated in miR - 300 - 3p - inhibited cells and reduced in miR - 300 - 3p - overexpressing cells (Figures 2E–G). These findings indicate that downregulation of miR - 300 - 3p promoted HepG2 cell lipid accumulation and hepatic inflammation.

### Downregulation of miR - 300 - 3p promotes apoptosis in HepG2 cells

**FIGURE 3:**
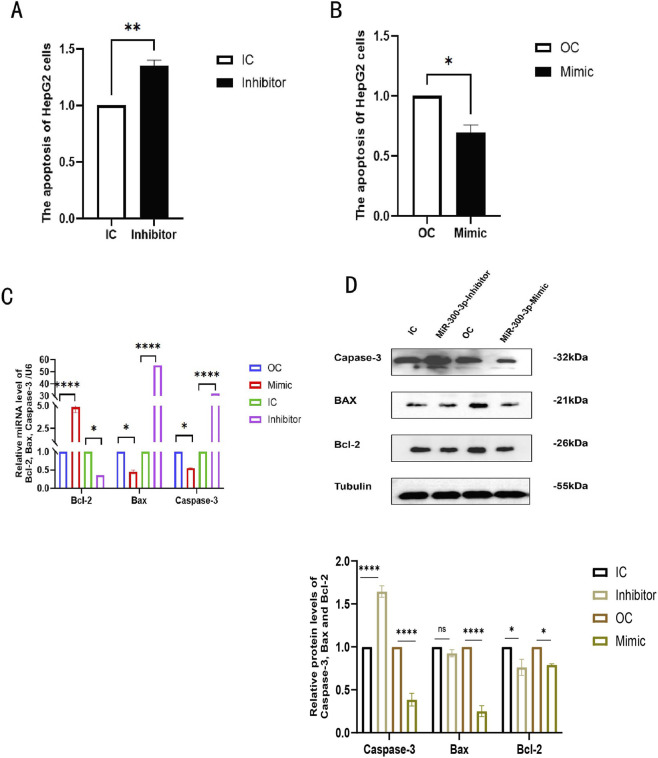
Downregulation of miR-300-3p induces apoptosis in HepG2 cells to promote it. **(A,B)** The apoptosis rate in miR-300-3p -inhibited and -overexpressing in HepG2 cells. **(C)** mRNA levels of Bax, Bcl-2 and Caspase-3 in miR-300-3p -inhibited and -overexpressing in HepG2 cells. **(D)** Protein levels of Bax, Bcl-2 and Caspase-3 in miR-300-3p -overexpressing and -inhibited in HepG2 cells. Data in **(A–D)** are means±SDs (n = 3). *P < 0.05, **P < 0.01, ***P < 0.001, ****P < 0.0001. OC, over-expression control; IC, inhibition control.

After treating the cells as shown in Figure 2A, we tested the apoptosis rate of each group and found the rate of apoptosis in HepG2 cells were increased in miR - 300 - 3p - inhibited HepG2 cells and declined notably in miR - 300 - 3p - overexpressing HepG2 cells (Figures 3A,B). At mRNA levels, Capase-3 and BAX expression was significantly increased in miR-300-3p-inhibited cells and decreased in miR - 300 - 3p - overexpressing cells, Bcl-2 expression produces the opposite result (Figure 3C). At the protein levels, Capase-3 was notably elevated in miR - 300 - 3p - inhibited cells and decreased in miR - 300 - 3p - overexpressing cells, Bcl-2 and BAX expression had not statistically significant difference in comparison with the control group (Figure 3D). Bcl-2 exhibits discordant expression patterns at the mRNA and protein levels. These discoveries suggest that downregulation of miR-300-3p promotes apoptosis in HepG2 cells.

#### miR - 300 - 3p targeted STX17, which is essential for the fusion of autophagosome and lysosome

**FIGURE 4:**
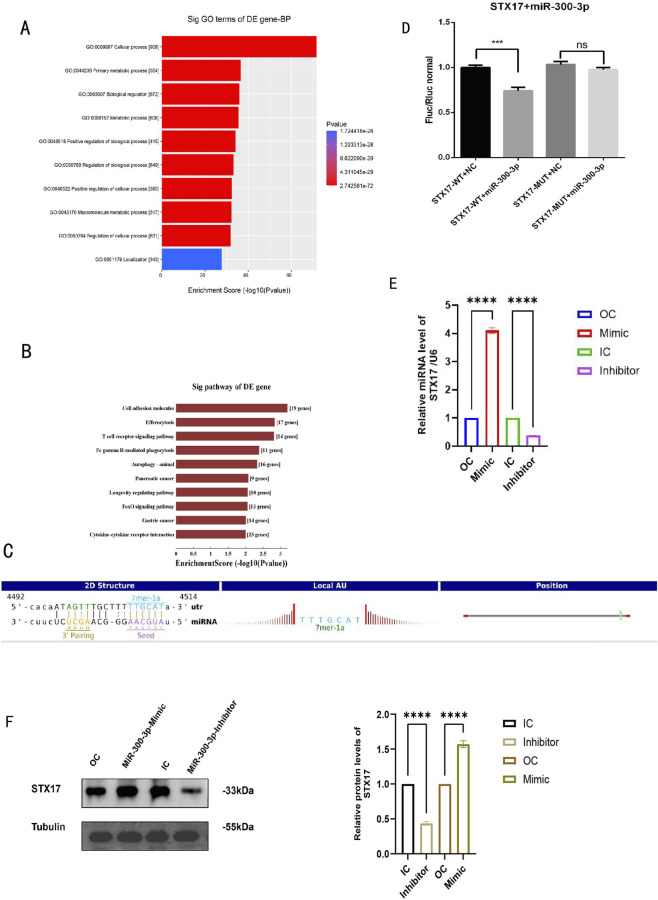
MiR-300-3p regulates STX17 in HepG2 cells. **(A,B)** GO enrichment analysis (Biological Process category) and KEGG enrichment analysis diagrams of sixteen predicted target genes of miR-300-3p. **(C)** The predicted binding sites between miR-300-3p and STX17 were putatively identified via the TargetScan database. **(D)** Dual-luciferase reporter assay (DLRA) in HepG2 cells validated that STX17 is a direct target gene of miR-300-3p. **(E,F)** The mRNA and protein expression levels of STX17 in HepG2 cells with miR-300-3p inhibition or overexpression. Data in **(A–D)** are means±SDs (n = 3). *P < 0.05, **P < 0.01, ***P < 0.001, ****P < 0.0001. OC, overexpression control; IC, inhibition control.

**FIGURE 5:**
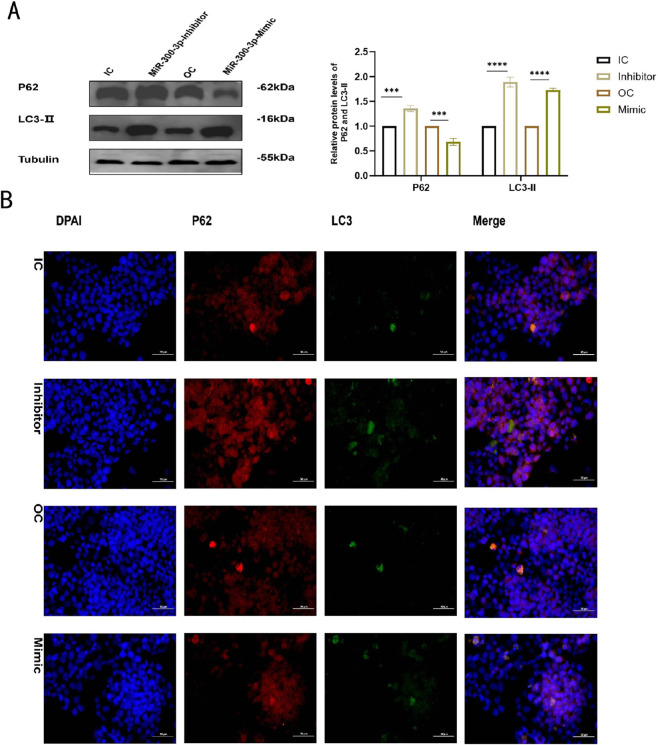
P62 and LC3-II in miR-300-3p -inhibited and -overexpressing in HepG2 cells. **(A)** The protein levels of P62 and LC3-II in miR-300-3p -inhibited and -overexpressing in HepG2 cells. **(B)** P62 and LC3-II in miR-300-3p -inhibited and -overexpressing by Immunofluorescence assay. Data in (A-D) are means±SDs (n = 3). *P < 0.05, **P < 0.01,***P < 0.001,****P < 0.0001. OC, overexpression control; IC, inhibition control.

The findings of the GO and KEGG analyses imply that miR - 300 - 3p participates in the autophagy process of MAFLD (Figure 4A). Sixteen genes, including STX17, were recognized as potential targets of miR - 300 - 3p (Figure 4B). STX17 is involved in the process of autophagy, which is essential for the fusion of autophagosome and lysosome. Predicted binding sites of miR - 300 - 3p and STX17 are shown in (Figure 4C). Firstly, the potential binding site of miR - 300 - 3p with the STX17 mRNA 3′-UTR was predicted, and the STX17 3^’^-UTR wild-type (WT) and STX17 3^’^-UTR mutant (MUT) sequence were cloned into the pmirGLO vector and co-transfected with miR - 300 - 3p into 293T cells for dual luciferase reporter assay. The relative expression levels of the STX17 -WT luciferase reporter gene showed a significant difference compared to the NC group (P < 0.05), indicating that miR - 300 - 3p exerts regulatory effects on STX17-WT in this experiment. Additionally, no significant difference was observed between the STX17 -MUT and the NC group (P > 0.05), suggesting that miR - 300 - 3p binds to the predicted binding site of STX17-WT to exert its regulatory function (Figure 4D). Dual-fluorescein enzyme labeling (DFE) in HepG2 cells confirmed that STX17 is a target of miR - 300 - 3p. The level of STX17 in HepG2 cells was markedly upregulated, which was completely consistent with the pattern of expression miR - 300 - 3p (Figure 4E). STX17 expression was significantly decreased in miR - 300 - 3p - inhibited HepG2 cells and markedly increased in overexpressing HepG2 cells (Figure 4F). The results was inconsistent with canonical miRNA biology predicts and this directional inconsistency suggests the relationship is more complex than a direct one-to-one repression, which indicated that the regulation of miR - 300 - 3p on STX17 might has context-dependent and involve indirect mechanism, feedback loop or multi-level control. Consistent with these findings, the downregulation of miR - 300 - 3p led to the aggregation of P62 and LC3-II, indicating the suppression of late - stage autophagy. Conversely, autophagy activation was detected in miR - 300 - 3p - overexpressing cells, as demonstrated by the enhanced degradation of p62 and the elevated levels of LC3-II (Figure 5A). Immunofluorescence assays corroborated the identical expression tendency (Figure 5B). These discoveries imply that miR - 300 - 3p directly interacts with STX17 and modulates its expression in HepG2 cells.

##### MiR - 300 - 3p promoted autophagy by regulating STX17, reduces lipid accumulation, inflammatory response, and decreases hepatocyte apoptosis

**FIGURE 6:**
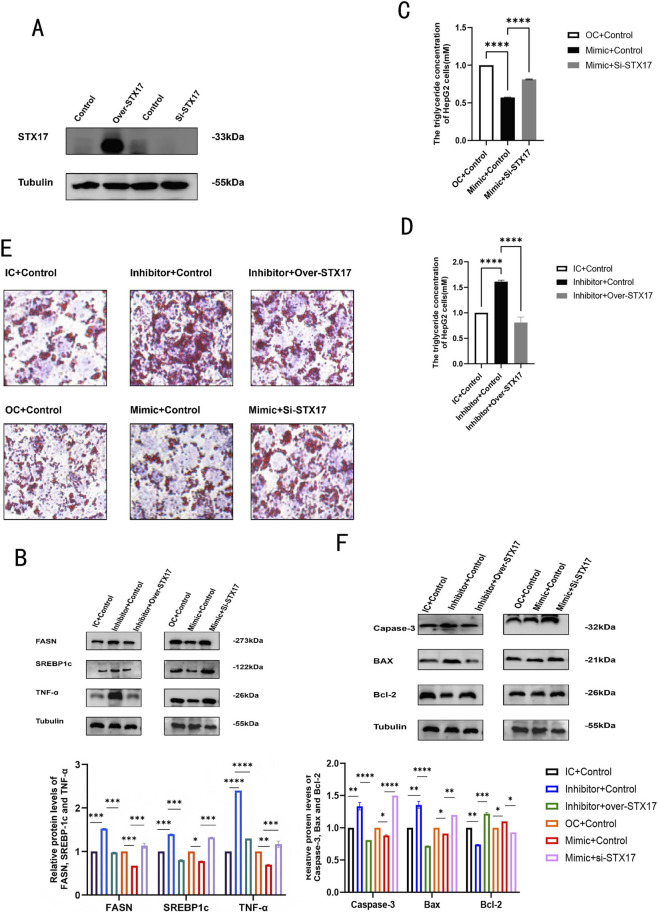
MiR-300-3p promoted autophagy by regulating STX17, reduces lipid accumulation, inflammatory response, and decreases hepatocyte apoptosis. **(A)** STX17 was inhibited or overexpressed in HepG2 cells successfully.**(B)** Protein levels of FASN, SREBP-1c and TNF-αin STX17 -inhibited and -overexpressing in HepG2 cells. **(C,D)** Intercellular TG contents in STX17-inhibited and -overexpressing in HepG2 cells. **(E)** Oil Red O staining (400×) and relative areas of lipid droplets in STX17-inhibited and -STX17 in HepG2 cells. **(F)** Protein levels of Bax, Capase-3 and Bcl-2 in STX17 -inhibited and -overexpressing in HepG2 cells. Data in **(A–D)** and **(F)** are means±SDs (n = 3). *P < 0.05, **P < 0.01, ***P < 0.001, ****P < 0.0001. OC, miR-300-3p overexpression control; IC,miR-300-3p inhibition control; Control, STX17-overexpression control or STX17-inhibition control; Si-STX17, STX17-inhibition; Over-STX17, STX17-overexpression.

**FIGURE 7:**
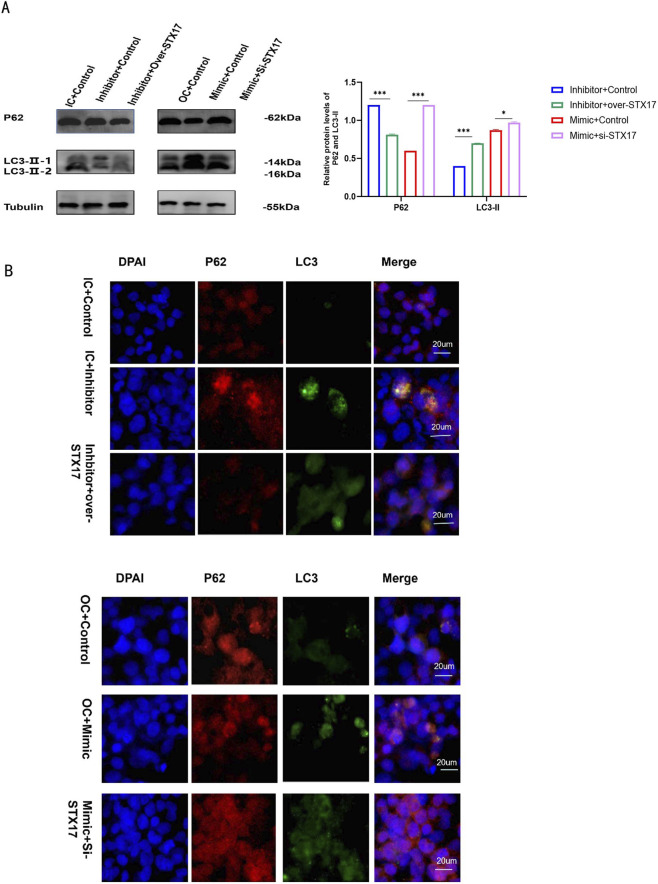
P62, LC3-II in STX17-inhibited and -overexpressing in HepG2 cells. **(A)** Protein levels of P62, LC3-II in STX17-inhibited and -overexpressing in HepG2 cells. **(B)** P62 and LC3-II in STX17-inhibited and -overexpressing by Immunofluorescence assay. Data in (A-D) are means±SDs (n = 3). *P < 0.05, **P < 0.01, ***P < 0.001, ****P < 0.0001. OC, overexpression control; IC, inhibition control.

STX17 was inhibited or overexpressed in HepG2 cells successfully (Figure 6A). After treating the cells as shown in the (Figure 2A). Previous experiments have found downregulation of miR - 300 - 3p led to the aggregation of P62 and LC3-II, showing the suppression of late-phase autophagy (Figure 5A). Subsequently, we assessed the capacity of STX17 to modulate lipophagy. In STX17-inhibited HepG2 cells, elevated levels of P62 and LC3 were observed, suggesting the suppression of autophagosome - lysosome fusion. Conversely, in STX17-overexpressed HepG2 cells, a reduction in P62 levels and an increase in LC3-II levels were detected, indicating a more active autophagy process (Figure 7A). Immunofluorescence assays confirmed the consistent expression trend (Figure 7B). In HepG2 cells, the suppression of STX17 expression led to a significant elevation in intracellular lipid droplet accumulation, triglyceride (TG) levels, and hepatic inflammatory responses, whereas STX17 overexpression exerted a pronounced inhibitory effect on these parameters (Figures 6B–D). At both the protein and mRNA levels, the expression of sterol regulatory SREBP-1c, FASN, and TNF-α was remarkably upregulated in STX17-silenced cells and notably downregulated in STX17-overexpressing cells (Figure 6B). The inhibition of STX17 expression promotes apoptosis in HepG2 cells. At the protein levels, Capase-3 and Bax was significantly increased in STX17-inhibited cells and decreased in STX17-overexpressed cells, Bcl-2 was the opposite result (Figure 6F). Consequently, we verified the association between the miR - 300 - 3p and STX17 signaling axes. Elevated STX17 levels facilitated the autophagy process, inhibited lipid accumulation and the inflammatory response, and reduced hepatocyte apoptosis. Consistent with this finding, when overexpressing miR - 300 - 3p, comparable effects were observed. These data indicated that miR - 300 - 3p and STX17 might be situated on the same signaling axis. Overexpression of STX17 inhibit steatosis-to-MASH progression.

### Clinical significance of miR - 300 - 3p in MASH patients

**FIGURE 8:**
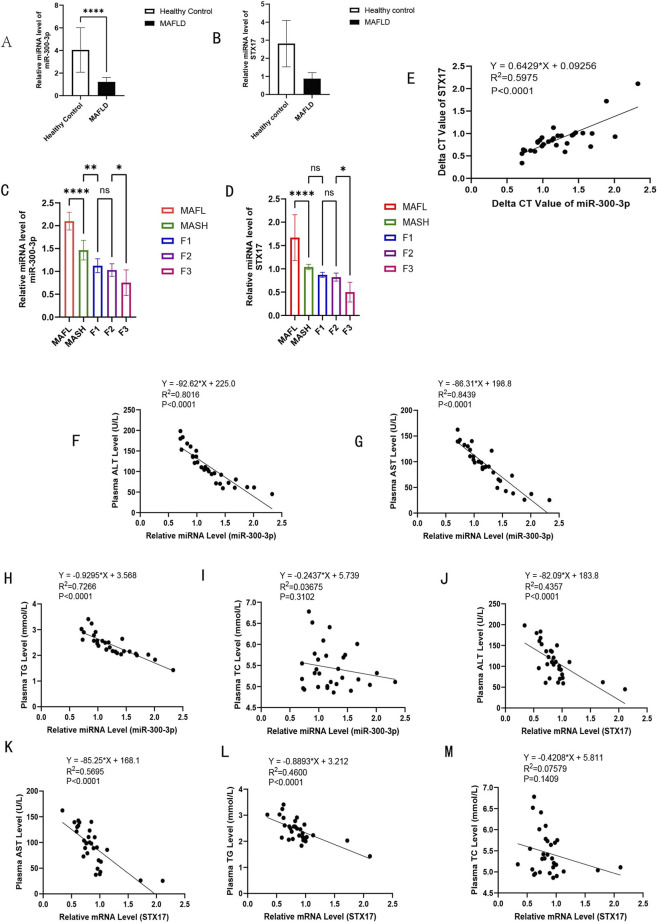
Clinical significance of miR-300-3p in MASH patients. **(A)** Relative miRNA level of miR-300-3p in the plasma of MAFLD patients, compared with normal healthy controls. **(B)** Relative mRNA level of STX17 in the plasma of MAFLD patients, compared with normal healthy controls. **(C)** Relative miRNA level of miR-300-3p in simple steatosis, MASH and in fibrosis grade. **(D)** Relative mRNA level of STX17 in simple steatosis, MASH and in fibrosis grade. **(E)** Correlation of delta CT value of miR-300-3p and STX17. **(F)** The plasma ALT levels were negatively correlated with the expression of miR-300-3p. **(G)** The plasma AST levels were negatively correlated with the expression of miR-300-3p. **(H)** The plasmaTG levels were negatively correlated with the expression of miR-300-3p. **(I)** No notable correlation was found between the expression of miR - 300 - 3p and TC levels. **(J)** The expression of STX17 presented a negative correlation with plasma ALT levels. **(K)** The expression of STX17 had a negative correlation with plasma AST levels. **(L)** The expression of STX17 had a negative correlation with plasma TG levels. **(M)** No significant correlation was obtained between STX17 expression with TC levels. *P < 0.05, **P < 0.01, ***P < 0.001, ****P < 0.0001.

We conducted an analysis of the potential clinical relevance of miR - 300 - 3p and STX17 in humans. We investigated the expression amounts of miR - 300 - 3p and STX17 in the plasma of thirty age-and sex-matched healthy individuals and liver biopsy-confirmed MAFLD patients (Supplementary 1). MAFL and MASH patients were differentiated based on MAS. Fibrosis scoring relies on the MAFLD guideline evaluation criteria (Eslam et al., 2025). The pathological report was issued by two professional pathologists separately. In comparison with the normal healthy control group, the levels of miR - 300 - 3p and STX17 in plasma of MAFLD patients were significantly reduced (Figures 8A,B). Compared with patients with simple steatosis, patients with MASH exhibited reduced levels of miR - 300 - 3p and STX17 in their plasma, and these levels gradually decreased as the degree of fibrosis increased (Figures 8C,D). Through linear regression analysis of ΔCT values (Li B. et al., 2023; Wang et al., 2013), we discovered that the expression of miR - 300 - 3p and STX17 was positively correlated in all subjects (Figure 8E). In addition, miR - 300 - 3p and STX17 were negatively associated with ALT and AST levels (Figures 8F,G,J,K). We also examined the relationship between miR - 300 - 3p and STX17 expression and lipid metabolism biomarkers. As a result, we found a significant and negative correlation of miR - 300 - 3p and STX17 expression with plasma total triglyceride concentrations (Figures 8H,L). However, no significant correlation was obtained between miR - 300 - 3p and STX17 expression with plasma total cholesterol concentrations (Figures 8I,M). In summary, these research findings recommend that the reduced expression level of miR - 300 - 3p may be associated with MASH and related lipid metabolism in human subjects.

## Discussion

In the current research, several dysregulated miRNAs were recognized between the two groups of mouse models via an unbiased screening of the normal - feeding and MAFLD mouse models. Our research shown the importance of miR - 300 - 3p in the progression of MAFLD. Firstly, miR - 300 - 3p expression remained downregulated in mouse models with MAFLD. Second, *in vitro* loss-of-function experiments demonstrated that knockdown of miR - 300 - 3p in steatotic HepG2 cells remarkably impaired cellular autophagic activity, promoted cell apoptosis, and triggered lipid accumulation, hepatic inflammation as well as hepatocellular injury-all of which represent core pathological hallmarks of MASH. In contrast, transduction of miR - 300 - 3p and over-STX17 successfully alleviated inhibit cell autophagy, cell apoptosis, lipid accumulation, liver inflammation and injury in steatotic HepG2 cells. We uncovered a miR- 300 - 3p - dependent pathway that regulates steatosis-to-MASH progression for the first time.

MicroRNAs (miRNAs) function as potent genetic regulators, capable of orchestrating entire cellular pathways through their interaction with a diverse array of target genes (Diener et al., 2022; Subramanian and Steer, 2019). This ability to modulate multiple components of a biological process makes miRNAs particularly attractive as therapeutic agents for restoring cellular functions that become dysregulated in disease states (Huang, 2017). Against this background, according to PubMed records, since 2010, over 1400 articles under the heading of ‘miRNA-based therapeutics’ have been published. Based on the findings of our research, liver-specific supplementation of miR - 300 - 3p might offer a potential option for therapeutic intervention against MASH.

We observed a notable elevation in the expression of Bcl-2 mRNA, while no corresponding alterations were detected in protein levels. This evident disparity is consistent with the well - established post - transcriptional regulatory networks. Amelia Cimmino et al. shown that Bcl-2 translation can be suppressed by miRNAs binding to its 3′UTR, blocking ribosomal assembly without mRNA decay (Cimmino et al., 2005). Sherrill KW reported the 5′UTR of Bcl-2 mRNA contains an internal ribosomal entry site (IRES), which selectively initiates translation under stress conditions independent of mRNA abundance (Sherrill et al., 2004). Zhong et al. (2005) found that the Bcl-2 protein can be targeted by E3 ubiquitin ligases and accelerated degradation through the ubiquitin-proteasome system (UPS). Under stress conditions, UPS activity may be enhanced, counteracting the protein accumulation caused by the upregulation of mRNA (Zhong et al., 2005). Our findings highlights the complexity of gene expression regulation and is not contradictory. The potential mechanisms underlying this phenomenon require further investigation in the future.

Through dual luciferase reporter assay and bioinformatic prediction showed that STX17 is a strong putative target of miR - 300 - 3p in MAFLD progression. Our manuscript reports that downregulation of miR-300-3p leads to decreased STX17 expression and promotes disease progression. However, canonical miRNA biology predicts that reduced miRNA levels should relieve repression and increase target gene expression (McGeary et al., 2019). The observed directionality (miR - 300 - 3p ↓ → STX17 ↓) contradicts established miRNA-mRNA regulation. Upon re-evaluating our data and the existing literature, this directional inconsistency suggests the relationship is more complex than a direct one-to-one repression. Many researches reported that miRNA regulation might has context-dependent and can involve indirect mechanisms, feedback loops, or multi-level control (Ren et al., 2025; Kalpachidou et al., 2025; Treekitkarnmongkol et al., 2026; Diener et al., 2024; Masalha et al., 2018; Hao et al., 2025). Emerging researchs highlight that miRNA-mediated regulation can exhibit bidirectional or paradoxical effects under pathological conditions (Diener et al., 2024). Vasudevan et al. substantiated that under the circumstances of cell cycle arrest or stress, miRNAs undergo a transformation from translation inhibition to translation activation (Vasudevan et al., 2007). Xiao et al. found that the miRNA - AGO2 complex is capable of entering the nucleus, binding to gene promoter regions, and directly initiating transcription (Xiao et al., 2017). The review clearly indicated that miRNAs indirectly modulate downstream genes by targeting transcription factors, thereby establishing a cascade effect. In this regard, the association between miRNAs and downstream genes might display a positive correlation (Bartel, 2009). Hao X et al. reported that CircMFN2/miR - 361 - 3p/ELK1 feedback loop promotes glutaminolysis and the progression of hepatocellular carcinoma (Hao et al., 2025). Building upon the aforementioned theoretical framework and the experimental data at hand, it is posited that the direct binding ability of miR - 300 - 3p to STX17 does not conflict with their positive correlation within endogenous cellular contexts. The former reflects their molecular recognition capability, whereas the latter reflects the biological reality wherein indirect transcriptional regulation predominates under MAFLD pathological conditions. This interpretation situates our findings within the advanced framework of miRNA regulatory networks and provides a clear direction for future investigations into the functional mechanisms of miR - 300 - 3p in MAFLD.

Our research reveals that alterations in STX17 mRNA and protein levels exhibit consistent trends under miR - 300 - 3p regulation. Furthermore, STX17 itself modulates the expression of transcription factors such as SREBP-1c and FASN. This suggests that STX17 likely resides within a complex transcriptional regulatory network, where its own expression is subject to feedback regulation. Clinical data further support the biological relevance of this model: in plasma samples from MAFLD patients, miR - 300 - 3p and STX17 levels show a positive correlation (Figure 6E), and both correlate with disease severity. This indicates that their positive association represents a genuine biological phenomenon in the pathological state *in vivo*. The observed regulatory patternc (miR - 300 - 3p↓ → STX17 ↓) represents a non-canonical yet biologically valid mode of regulation. This non-canonical interaction arises from the synergistic effects of direct binding between miR - 300 - 3p and STX17 and transcriptional feedback mediated by the autophagy pathway. *In vivo*, *in vitro*, and clinical evidence collectively corroborate the biological validity of the miR - 300 - 3p/STX17 axis in the progression of MAFLD. However, the underlying mechanism through which miR - 300 - 3p regulates STX17 requires further investigation.

Macroautophagy (hereafter referred to as autophagy) involves the deterioration of cellular components through the combination of autophagosomes and lysosomes (Zadoorian et al., 2023; Zhang et al., 2018). It is well-recognized for its function in regulating the progression of MAFLD (Rho et al., 2024). The SNAP29-STX17-VAMP8 SNARE complex facilitates autophagosome-lysosome membrane blend. STX17 is situated on the autophagosome membrane, interacts with the adaptor protein SNAP29, which subsequently associates with VAMP8 anchored on the lysosomal membrane (Wen-You Yim and Mizushima, 2020; Itakura et al., 2012). STX17 has been relatively more extensively studied. Cheng X et al. demonstrated that hepatic deletion of Rubcnl disrupts the association between RUBCNL and STX17, as well as the complex encompassing the transmembrane and ubiquitin-like domain 1. Consequently, autophagosome maturation is abolished, leading to lipid accumulation and liver fibrosis in a mouse model of MAFLD under nutrient - rich conditions (Ren et al., 2024; Li Y. et al., 2023). Tripathi et al. (2022) has shown the proteasomal degradation of STX17, which is promoted by increased homocysteinylation and ubiquitination, plays a part in the advancement of MALFD (Tripathi et al., 2022). Additionally, increased acetylation of PACER triggers an interaction of the HOPS complex with STX17. Furthermore, the deficiency of SRSF3 results in ubiquitination-dependent proteasomal degradation of STX17, thereby reducing lipophagy under conditions characterized by an abundance of free fatty acids (FFAs) (Cheng et al., 2019; Li Y. et al., 2023). The enhancement of autophagy through pharmacological methods has been demonstrated to mitigate liver steatosis and injury in MAFLD in pre -clinical models (Byrnes et al., 2022; Lin et al., 2013; Ding et al., 2010). Therefore, therapeutic targeting of autophagy may hold therapeutic potential for the management of metabolic liver diseases. In the present study, our findings indicated that aberrant expression of STX17 triggered cell apoptosis, lipid accumulation, liver inflammation and injury in steatotic HepG2 cells. This finding is consistent with the effects of miR - 300 - 3p deficiency. The promotional role of STX17 in the progression of MASH was further verified in MASH patients. These findings imply that STX17 was extensively implicated in the pathogenesis of MASH. There is currently limited research on miR - 300 - 3p. Furthermore, Jinlian Liang et al. have shown that miR - 300 - 3p is associated with testosterone deficiency in male obesity, and inhibiting miR - 300 - 3p can effectively alleviate the symptoms of testosterone deficiency in obese mice. These findings suggest that miR - 300 - 3p plays a crucial role in the differentiation and function of Leydig cells (LC), and may serve as a potential diagnostic or therapeutic target for obesity-related testosterone deficiency (Liang et al., 2023). Yan -na et al. has shown that miR-300-3p can alleviate hypoxia/reoxygenation-induced H9c2 cardiomyocyte injury by regulating autophagy (Yan -na et al., 2022). Nevertheless, considering that a single miRNA typically has the ability to target multiple mRNAs, additional investigations are required to pinpoint more targets of miR - 300 - 3p in metabolic liver diseases.

In conclusion, sufficient evidence backs the function of miRNAs in the pathophysiological processes behind the development of MAFLD. MiR-34a, miR-21, and miR-122 are also among the most comprehensively studied miRNAs in regulating the molecular pathways involved in the occurrence and development of MAFLD (Guo et al., 2016; Liu et al., 2018; Rui et al., 2013; Xu et al., 2021; Miyaaki et al., 2014; Jiang et al., 2013). Numerous studies have investigated rodent experimental models of MAFLD or MASH, yet research assessing miRNA expression changes in human MAFLD has been relatively scarce (Hochreuter et al., 2022). Thus, there are at least two advantages of miR - 300 - 3p. First, Our study demonstrated miR - 300 - 3p inhibits the progression of MAFLD by regulating autophagy, influencing apoptosis, lipid metabolism, and inflammation at both cellular and population levels. Second, evidence collectively corroborate the biological validity of the miR - 300 - 3p/STX17 axis in the progression of MAFLD. Our findings regarding miR - 300 - 3p have important clinical significance. This study has several limitations. Primarily, while HepG2 cells provide a well-established model for hepatic studies, the absence of validation in primary rat hepatocytes or *in vivo* models limits direct extrapolation to physiological systems. Future studies will address this limitation through complementary animal models. Secondly, the human component (n = 60) serves as preliminary translational evidence. Larger cohort studies with tissue-level analysis are warranted to confirm these clinical associations. Finally, the more in - depth mechanisms by which miR - 300 - 3p regulates the expression of STX17 necessitate further investigation.

## Data Availability

The data presented in the study are deposited in the NCBI GEO repository, accession number GSE328114.
